# Prevalence and diversity of avian blood parasites in a resident northern passerine

**DOI:** 10.1186/s13071-019-3545-1

**Published:** 2019-06-10

**Authors:** Caroline Van Hemert, Brandt W. Meixell, Matthew M. Smith, Colleen M. Handel

**Affiliations:** U.S. Geological Survey, Alaska Science Center, 4210 University Dr., Anchorage, AK 99508 USA

**Keywords:** Haemosporidian parasites, Alaska, Northwestern crow, *Corvus caurinus*, *Plasmodium*, *Haemoproteus*, *Leucocytozoon*, Co-infection, Avian keratin disorder

## Abstract

**Background:**

Climate-related changes are expected to influence the prevalence and distribution of vector-borne haemosporidian parasites at northern latitudes, although baseline information about resident birds is still lacking. In this study, we investigated prevalence and genetic diversity of *Plasmodium*, *Haemoproteus,* and *Leucocytozoon* parasites infecting the northwestern crow (*Corvus caurinus*), a non-migratory passerine with unique life-history characteristics. This species occupies both intertidal and forested habitats and is subject to high prevalence of avian keratin disorder (AKD), a disease that causes gross beak deformities. Investigation of avian blood parasites in northwestern crows at sites broadly distributed across coastal Alaska provided an opportunity to evaluate specific host factors related to parasite infection status and assess geographical patterns of prevalence.

**Results:**

We used molecular methods to screen for haemosporidian parasites in northwestern crows and estimated genus-specific parasite prevalence with occupancy modeling that accounts for imperfect detection of parasite infection. We observed considerable geographical and annual variation in prevalence of *Plasmodium*, *Haemoproteus*, and *Leucocytozoon*, but these patterns were not correlated with indices of local climatic conditions. Our models also did not provide support for relationships between the probability of parasite infection and body condition or the occurrence of co-infections with other parasite genera or clinical signs of AKD. In our phylogenetic analyses, we identified multiple lineages of each parasite genus, with *Leucocytozoon* showing greater diversity than *Plasmodium* or *Haemoproteus*.

**Conclusions:**

Results from this study expand our knowledge about the prevalence and diversity of avian blood parasites in northern resident birds as well as corvids worldwide. We detected all three genera of avian haemosporidians in northwestern crows in Alaska, although only *Leucocytozoon* occurred at all sites in both years. Given the strong geographical and annual variation in parasite prevalence and apparent lack of correlation with climatic variables, it appears that there are other key factors responsible for driving transmission dynamics in this region. Thus, caution is warranted when using standard climatic or geographical attributes in a predictive framework. Our phylogenetic results demonstrate lower host specificity for some lineages of *Leucocytozoon* than is typically reported and provide insights about genetic diversity of local haemosporidian parasites in Alaska.

**Electronic supplementary material:**

The online version of this article (10.1186/s13071-019-3545-1) contains supplementary material, which is available to authorized users.

## Background

Blood parasites of the order Haemosporidia are vector-borne parasites that infect amphibians, reptiles, birds, and mammals worldwide [1]. Three genera of haemosporidians cause malaria and malaria-like diseases in birds: *Plasmodium* is transmitted by mosquitoes (Culicidae), *Haemoproteus* is transmitted by biting midges (Ceratopogonidae), and *Leucocytozoon* is transmitted by black flies (Simuliidae) [1]. The impacts on fitness of birds from haemosporidian infections vary widely, ranging from no or only mild clinical effects among species that have coevolved with these parasites [2–4] to severe morbidity or mortality among previously unexposed individuals [5]. In some instances, introduction of blood parasites to naïve bird populations may contribute to large-scale declines or extinctions, such as those that have occurred among certain endemic Hawaiian land birds in response to *Plasmodium* infection [6–8]. Other observed effects of infection, such as reduced reproductive success, compromised immune response, and increased senescence [9, 10], may be subtler but still result in substantial long-term demographic consequences.

Many biotic and abiotic factors can influence the distribution and prevalence of avian haemosporidian parasites, but climatic variables such as temperature are thought to play a key role in the dynamics of transmission [9, 11–13]. As such, there is growing concern regarding the potential effects of climate change on avian malaria and malaria-like diseases in wild birds, particularly in northern regions where the effects of warming are most pronounced [14]. Once thought to be largely absent from the Arctic and sub-Arctic, avian blood parasites are now known to occur across broad habitat, climatic, and latitudinal gradients. *Leucocytozoon* has been detected in birds throughout temperate, boreal, and Arctic regions of North America, often at relatively high prevalence in both adult and juvenile birds, demonstrating local transmission and completion of the parasite life-cycle [15–18]. Although *Haemoproteus* and *Plasmodium* parasites are also known to infect birds in northern regions, infections by these genera typically occur at lower frequency and the northern limit of their transmission appears to be geographically restricted to habitats within the sub-Arctic [15, 16, 18, 19]. To date, there is no direct evidence that *Haemoproteus* or *Plasmodium* parasites are transmitted in the North American Arctic [17, 18, 20, 21]. A number of studies have hypothesized that changes to the spatial distribution of haemosporidian parasites or their vectors may occur in response to climate warming in the Arctic and sub-Arctic [21–23]. However, baseline information on the distribution and prevalence of haemosporidian parasites is lacking for many resident avian species, thus making it difficult to assess current infection status and track future changes.

The northwestern crow (*Corvus caurinus*) is a coastally distributed year-round resident of the Pacific Northwest region of North America. This species occupies a unique ecological niche among passerines in its reliance on marine intertidal resources for much of the year [24]. Although northwestern crows are omnivorous and opportunistic consumers that nest in forested habitats, they typically occur in close proximity to the coast [24] and thus encounter a diversity of potential vectors in both terrestrial and nearshore marine habitats. Previous studies have suggested that members of the corvid family (Corvidae) may serve as important reservoirs or hosts for maintaining avian blood parasites [25, 26]. Crows, like other corvids, are synanthropic and tend to congregate near human settlements, which may also influence their exposure to vectors [27]. Additionally, northwestern crows are susceptible to avian keratin disorder (AKD), a disease of wild birds that results in gross beak deformities and other abnormalities of keratinized tissues [28, 29]. In a diverse array of avian species, the status of infection by haemosporidian parasites has been associated with characteristics of the individual host, including age [30, 31], sex [32, 33], body condition [34, 35], and co-occurrence of other diseases [4, 16, 19, 36–39]. Specifically, compromised immune function or other physiological or behavioral changes related to a disease such as AKD may affect an individual’s susceptibility to other infections. For instance, a recent study of black-capped chickadees (*Poecile atricapillus*) affected by AKD determined that birds with beak deformities were 2.6 times more likely to be infected with *Plasmodium* than those with normal beaks [19]. In some instances, an inverse relationship between avian malaria and other diseases, such as West Nile virus, has been observed [36]. Among corvids, there is some evidence that *Plasmodium* can have pathogenic effects. Impacts on individual survival and fitness were first observed in Hawaiian crows (*C. hawaiiensis*) that may have evolved in isolation from parasites of this genus [6, 9, 40], but have also recently been documented among nestling American crows (*C. brachyrhynchos*) in California, where avian populations have likely had historical exposure to *Plasmodium* [41].

In this study, we evaluated the prevalence and genetic diversity of *Plasmodium, Haemoproteus,* and *Leucocytozoon* parasites infecting northwestern crows over a broad geographical area of southcentral and southeastern Alaska. To identify possible drivers and potential consequences of blood parasite infection in this species, we examined the (i) effects of location, year, and climatic and host factors on parasite prevalence; (ii) patterns of co-infection among the parasite genera and with AKD; and (iii) relationships between body condition and parasite infection. We also investigated phylogenetic relationships of *Plasmodium*, *Haemoproteus*, and *Leucocytozoon* parasites in northwestern crows relative to other Alaskan avian hosts and corvids from around the world. Our results provide baseline information about haemosporidian parasites in an ecologically unique northern passerine and expand knowledge about parasite diversity and host specificity among corvids.

## Methods

### Sample collection

**Fig. 1:**
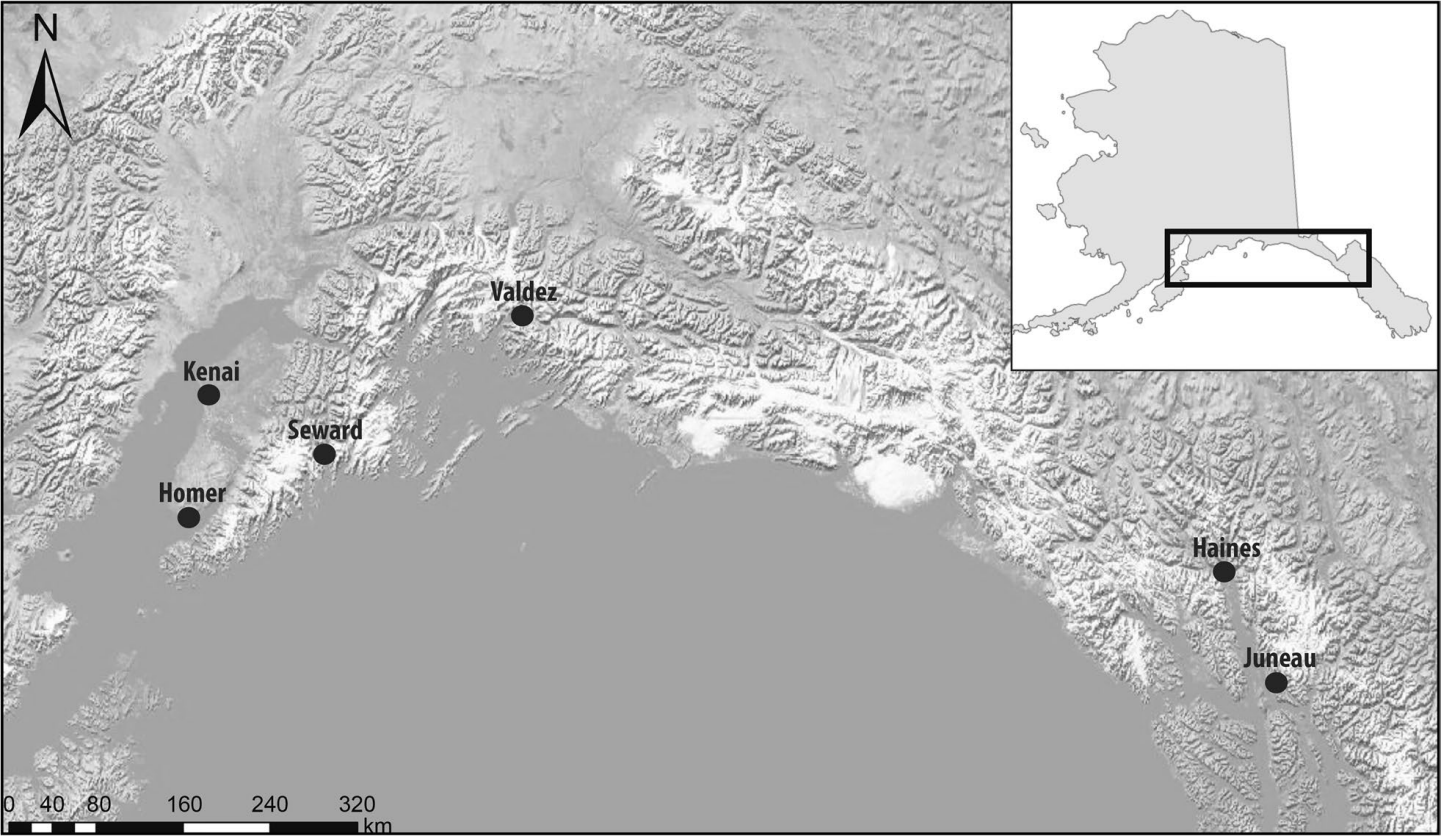
Map of sampling locations for northwestern crows captured in southcentral and southeastern Alaska during 2007 and 2008

During February–April of 2007 and 2008, we sampled 186 non-molting northwestern crows at six sites in southcentral and southeastern Alaska (Fig. 1). We captured birds at Seward (60.11°N, 149.44°W), Kenai (60.55°N, 151.23°W), and Homer (59.64°N, 151.54°W) in 2007 and 2008 and at Valdez (61.12°N, 146.35°W), Haines (59.23°N, 135.44°W), and Juneau (58.38°N, 134.64°W) in 2008 using modified drop-net traps [42, 43] and bungee-loaded whoosh nets [29, 44]. All sampling locations were characterized by coniferous forest adjacent to tidally affected coastal habitat. We identified birds as juveniles (< 1 year of age) or adults (≥ 1 year) based on molt limits, rectrix shape, and mouth color [45]. We weighed birds to the nearest gram using a digital scale and measured tarsus length (± 0.1 mm) and culmen length (± 0.1 m) using digital calipers and wing length (± 1 mm) using a metal ruler. We collected 0.5–1.0 ml of whole blood from the brachial vein and preserved samples in Longmire buffer prior to analysis [46]. We sexed birds molecularly from blood samples and classified birds as affected or unaffected by AKD based on the presence or absence of a beak deformity per criteria described in Van Hemert & Handel [29].

### Haemosporidian detection

We extracted DNA from blood samples using the DNeasy Blood and Tissue Kit (Qiagen, Valencia, CA) following the manufacturer’s protocol. Each sample was simultaneously screened for *Plasmodium*, *Haemoproteus*, and *Leucocytozoon* blood parasites using a nested-PCR method described by Hellgren et al. [47], which amplifies a 479 bp fragment of the parasite’s mitochondrial DNA (mtDNA) cytochrome *b* (*cytb*) gene. To be able to account for imperfect detection of infections when modeling prevalence [48, 49], we screened all samples twice, visualizing them on 0.8% agarose gels stained with Gel Red Nucleic Acid Gel Stain (Biotium, Hayward, CA). We purified positive PCR products with Exo-SapIT (USB Inc., Cleveland, OH) using a 3:7 dilution with ultra-pure water and following the manufacturer’s recommended cycling protocol. Sequencing was conducted using identical primers as PCR and Big Dye Terminator v3.1 mix and analyzed on an ABI 3730xl automated DNA sequencer (Applied Biosystems, Foster City, CA). Raw sequences were edited with Sequencher 5.0.1 software (Gene Codes Corp., Ann Arbor, MI) and assigned to one of three parasite genera (*Plasmodium*, *Haemoproteus*, or *Leucocytozoon*) using the nucleotide BLAST function available on the National Center for Biotechnology Information (NCBI) website. We considered samples to be negative on a given PCR reaction if they did not produce a double-stranded DNA sequence or if they failed to be assigned *via* BLAST.

### Statistical analysis

The probability of detecting haemosporidian mtDNA in avian blood samples using molecular methods is generally high, but less than perfect, typically ranging between 0.75 and 0.95 [16, 50, 51]. The sensitivity of this test is likely associated with the parasite load in the host [48, 50], and can also vary relative to parasite genus and a number of host characteristics, including species, age, and sex [16, 18, 52]. When unaccounted for, imperfect detection of parasite infection (i.e. occurrence of false negatives) may result in biased estimates of prevalence [49]. Therefore, we used occupancy modeling with replicate analyses of blood samples to obtain estimates of parasite prevalence adjusted for false negatives in the dataset [48]. More specifically, occupancy models allow for simultaneous estimation of the probability of infection (Ψ, prevalence) and the probability of detecting parasite mtDNA within a sample, given that mtDNA was present (p, detection probability; [53]). We conducted occupancy modeling using Program MARK [54] and report estimates ± standard error (SE) unless otherwise stated.

To characterize the epidemiology of haemosporidian infection in northwestern crows, we developed an *a priori* suite of models to assess variation in parasite detection and prevalence relative to several temporal, spatial, and host-specific characteristics. We conducted model selection using a staged approach to limit the total number of models considered, and we assessed relative support among models using Akaike’s information criterion corrected for sample size (AIC_c_; [55]). Analyses for each parasite genus were conducted separately, following the same general approach.

#### Stage 1 (general patterns of variation in detection and prevalence)

In Stage 1 of model selection, we considered two suites of 20 models to assess variation in (i) detection probability and (ii) prevalence, testing each parameter relative to combinations of sampling location, year, and age and sex of the crows. Each suite contained models with each variable alone and all additive combinations of each variable; it also included models with a location × year interaction with and without the additive effects of age and sex (Additional file 1: Table S1). We first assessed potential sources of variation on detection probability by constraining prevalence to a relatively complex structure (i.e. site + year + sex) while considering each combination of the factors above on detection probability. We then fixed detection probability to the top-supported structure and considered the same combinations of factors on prevalence. The top approximating model from Stage 1 was then used as the base structure in subsequent stages of analysis. Because *Haemoproteus* infections were detected only at the sites that were sampled in a single year, we did not consider models containing annual variation for this parasite.

#### Stage 2 (body condition)

To assess a potential relationship between individual host condition and probability of infection with blood parasites, we considered a suite of models in Stage 2 that assessed variation in infection status relative to a body condition index (BCI) representing size-adjusted body mass. We surmised that a correlation between body condition and blood parasite infection could result from one of two possible scenarios: (i) *Plasmodium*, *Haemoproteus*, or *Leucocytozoon* imparts fitness consequences on infected individuals; or (ii) reduced condition increases an individual’s susceptibility to infection or recrudescence of latent infections (sensu [56]). We created the BCI variable by performing a principal components analysis on the correlation matrix for lengths of tarsus and wing, regressing body mass on the first principal component score (PC1; linear combination of both tarsus and wing that corresponded to the overall size of the bird), and then dividing residuals from the regression by mean mass. Because body size and mass vary by age and sex in northwestern crows [24], we controlled for this variation by calculating BCI separately for each age and sex class. We then combined the class-specific indices into a single variable for inclusion in the occupancy analysis, allowing us to assess the relative relationship between body condition and infection status in a single parameter [16]. For each parasite genus, we considered a model in which prevalence was allowed to vary by BCI alone, by the top-supported structure from Stage 1 with an additive effect of BCI, and by a BCI effect on each source of variation in the top Stage 1 model.

#### Stage 3 (co-infections)

Next, we assessed whether infection with a given parasite genus was dependent on infection by another parasite genus or with clinical signs of AKD (hereafter, defined as “co-infection”). Although co-infections were relatively common in our dataset, some combinations of co-infection were detected at only a subset of study sites. Therefore, in each analysis, we partitioned the data to include only the study sites for which the 2 states in question were both detected. For example, when modeling prevalence of *Haemoproteus* relative to co-infection with *Leucocytozoon*, we limited the data to only blood samples obtained at Haines and Juneau because infection with *Haemoproteus* parasites was rare or not detected at the other sites. Variables for infection by each parasite genus and for clinical signs of AKD were coded as individual binomial covariates. For each pairwise combination we considered models in which Ψ was constant for the parasite of interest or was allowed to vary by the other co-infection variable, by the top-supported structure from Stage 2 with an additive effect of the co-infection variable, and by a co-infection effect on each source of variation in the top Stage 1 model. When assessing relationships between parasite infection and AKD, we partitioned the data to include only adults because AKD occurs almost exclusively in adults [29] and none of the juvenile crows in our sample had clinical signs of AKD. We did not detect any co-infections containing *Plasmodium* and *Haemoproteus* parasites and therefore did not consider models assessing this co-infection relationship.

#### Stage 4 (weather variables)

In the final modeling stage, we examined relationships between parasite infection status and several weather variables recorded for each combination of site and year. At northern latitudes, haemosporidian parasite transmission is thought to occur primarily during summer months when suitable vectors are active [1, 57]. Hence, although blood samples were collected from crows during winter months, we expected environmental conditions during the previous summer to best explain spatial and annual patterns of variation in prevalence [19]. As such, we constructed year-specific weather indices over the period of 1 May–31 August that corresponded with the summer preceding crow captures at each site in each year. We obtained site-specific data for constructing weather indices using NOAA online data tools (https://www.ncdc.noaa.gov/cdo-web/datatools/lcd); weather stations were located within 2–6 km of our capture sites. We selected three weather variables for consideration: (i) average hourly temperature (temp; °C); (ii) average hourly wind speed (wind; km/h); and (iii) cumulative precipitation (precip; cm). We expected genus-specific prevalence to be positively correlated with temperature and precipitation because these conditions favor reproduction of both vectors and parasites [11, 12, 58]. Vector activity, and hence the ability to transmit parasites, is likely reduced by windy conditions [59]. Therefore, we expected to find a negative relationship between wind speed and prevalence. For *Leucocytozoon* parasites, which were detected in blood samples at all locations in both years, we considered a suite of 6 models in which site and year were replaced with each weather variable alone, and in two-way additive combinations. Because *Plasmodium* and *Haemoproteus* parasites were detected in crows at only a limited number of sites, we were unable to directly model relationships between weather variables and prevalence; therefore, we drew inference about these potential relationships by comparing site-specific weather indices between sites for which these parasites were detected and sites where prevalence was zero.

### Phylogenetic analysis

We performed phylogenetic analyses on haemosporidian mtDNA sequences to assess patterns of geographical or host specificity. We included a single representative of each unique parasite mtDNA lineage identified in our study (*n* = 10; GenBank: MG765392-MG765401) along with reference sequences obtained from GenBank and MalAvi databases [60]. Reference sequences included lineages previously detected in Alaskan birds [15, 17, 61] as well as representative lineages from corvid species as reported on the MalAvi and GenBank databases. We aligned all sequences and cropped them to a final length of 425 bp; any lineages that were shorter or contained ambiguous bases were excluded from further analysis. We constructed Bayesian phylogenies using MrBayes 3.2.5 [62] using a general time-reversible model and a gamma distribution for among-site variation (GTR + G). Trees were sampled every 1000 generations with the first 25,000 generations being discarded as ‘burn-in.ʼ Each analysis was run for a minimum of 3.0 × 10^6^ generations or until the split frequencies of the posterior probability’s standard deviation was less than 0.01 and trees were rooted with *Leucocytozoon* sequences based on Lutz et al. [63].

## Results

### Prevalence

We detected avian blood parasites in 129 of the 186 northwestern crows sampled; apparent prevalence of parasite infections was 9.7% for *Plasmodium*, 29.6% for *Haemoproteus*, and 53.8% for *Leucocytozoon* (Table 1). Presence of *Plasmodium* and *Haemoproteus* showed considerable spatial variation, with each genus being detected in crows at only 3 sites, respectively. In contrast, *Leucocytozoon* infections occurred at all study sites in both years. Co-infections between parasite genera were relatively common, with 34 co-infections of *Haemoproteus* and *Leucocytozoon*, and 9 co-infections of *Plasmodium* and *Leucocytozoon.* No co-infections of *Plasmodium* and *Haemoproteus* were detected, which could be attributed to the fact that the nested PCR protocol designed by Hellgren et al. [47] uses a single primer pair for amplifying *Plasmodium* and *Haemoproteus* DNA, making co-infections between these two genera difficult to detect [64]. Nineteen adult crows showed signs of AKD infection; 12 AKD infections were associated with co-infections by at least one parasite genus including *Plasmodium* (*n* = 3), *Haemoproteus* (*n* = 3) and *Leucocytozoon* (*n* = 9). Co-infections involving AKD, *Plasmodium*, and *Leucocytozoon* were detected in 2 crows, and a single crow was infected with AKD, *Haemoproteus*, and *Leucocytozoon*.

**Table 1:** Occurrence of *Plasmodium* (Plas), *Haemoproteus* (Haem), and *Leucocytozoon* (Leuc) parasites in northwestern crows (*Corvus caurinus*) at six sites in coastal Alaska sampled during 2007 and 2008. Columns show number of individuals with a positive result on one or more PCR runs and apparent prevalence (in %, in parentheses), uncorrected for imperfect detection

Location	Year	Month	*n*	Plas-positive	Haem-positive	Leuc-positive
Kenai	2007	March	9	6 (66.7)	0	6 (66.7)
	2008	February–March	15	7 (46.7)	0	3 (20.0)
Homer	2007	March	10	2 (20.0)	0	10 (100)
	2008	Feb	22	2 (9.1)	2 (9.1)	17 (77.3)
Seward	2007	March	8	0	0	3 (37.5)
	2008	Feb	20	0	0	9 (45.0)
Valdez	2008	March	37	0	1 (2.7)	11 (29.7)
Haines	2008	April	30	1 (2.9)	25 (71.4)	26 (74.3)
Juneau	2008	April	35	0	29 (96.7)	15 (50.0)
Total			186	18 (9.7)	55 (29.6)	100 (53.8)

**Fig. 2:**
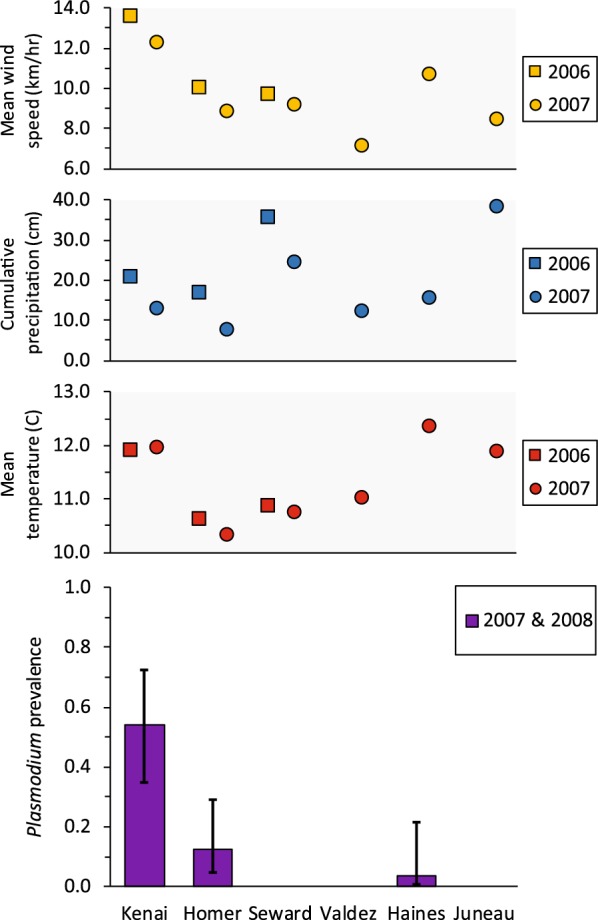
Estimated prevalence (± 95% CI) of *Plasmodium* parasites infecting northwestern crows (*Corvus caurinus*) sampled in late winter during 2007 and 2008 (purple; combined estimate) at 6 sites in southcentral and southeastern Alaska. Mean hourly wind speed (yellow), cumulative precipitation (blue), and mean daily temperature (red) for summer (1 May to 31 August) during the preceding year are shown with a square (2006) or circle (2007). No relationships were apparent between parasite prevalence and any of the weather variables. Note that Valdez, Haines, and Juneau were sampled in 2008 only

The distribution of infections by *Plasmodium* parasites showed strong spatial variation, with an apparent lack of infections at some sites. *Plasmodium* infections were identified in both years at Kenai and Homer, but there were no infections in either year at Seward. For samples obtained from sites in 2008 only, we detected one *Plasmodium* infection at Haines, but no infections at Valdez or Juneau. There were no discrepancies among replicate PCR runs of blood samples containing *Plasmodium* mtDNA and therefore detection probability was 1.0. The top supported Stage 1 model allowed prevalence to vary by site; we found only limited support for the effects of age (ΔAIC_c_ = 0.38; higher for adults) and year (ΔAIC_c_ = 0.58; higher in 2007) on prevalence (Additional file 1: Table S2). Estimated *Plasmodium* prevalence was 0.54 (± 0.10) at Kenai, 0.13 (± 0.06) at Homer, 0.04 (± 0.04) at Haines, and 0 at remaining sites (Fig. 2). We found no support for effects of body condition or co-infections on *Plasmodium* prevalence (Additional file 1: Table S2). We were unable to directly model relationships between weather variables and *Plasmodium* prevalence due to the sporadic spatial occurrence of *Plasmodium* infections, but visual inspection of plots of wind, temperature, and precipitation against *Plasmodium* prevalence did not suggest any clear relationships (Fig. 2).

**Fig. 3:**
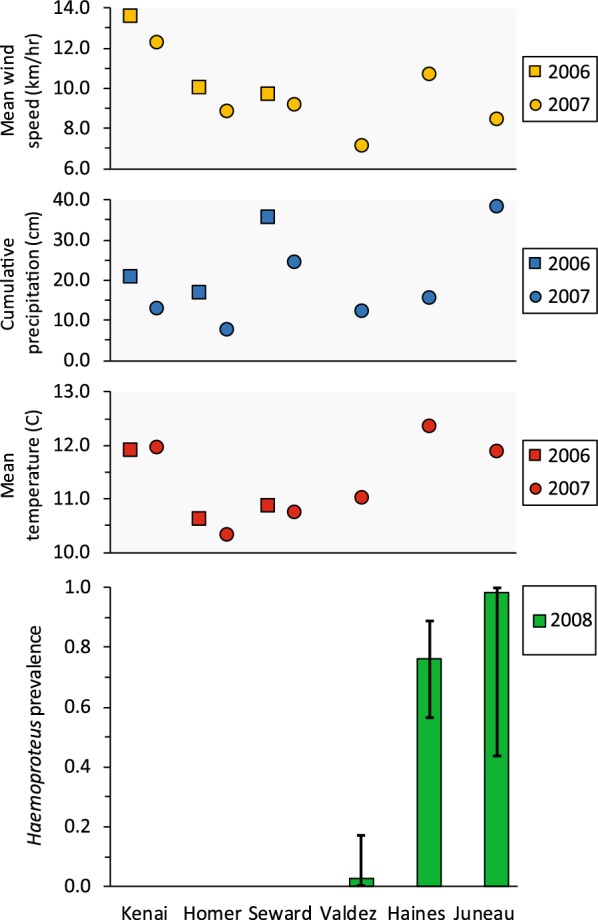
Estimated prevalence (± 95% CI) of *Haemoproteus* parasites infecting northwestern crows (*Corvus caurinus*) sampled in late winter during 2007 and 2008 (green) at 6 sites in southcentral and southeastern Alaska. There were no detections of *Haemoproteus* parasites during 2007. Mean hourly wind speed (yellow), cumulative precipitation (blue), and mean daily temperature (red) for summer (1 May to 31 August) during the preceding year are shown with a square (2006) or circle (2007). No relationships were apparent between parasite prevalence and any of the weather variables. Note that Valdez, Haines, and Juneau were sampled in 2008 only

*Haemoproteus* infections were common in crows sampled at the 2 sites in southeastern Alaska (Haines and Juneau) during 2008, but only a single infection was identified among samples from the remaining sites and years (Table 1, Fig. 3). The top approximating Stage 1 model for *Haemoproteus* estimated a constant detection probability of 0.87 (± 0.04) and allowed prevalence to vary by location (Additional file 1: Table S3). There was limited support for a negative effect of age on *Haemoproteus* detection (ΔAIC_c_ = 1.21), but no support for variation in detection relative to any other variables (Additional file 1: Table S3). Estimated *Haemoproteus* prevalence was 0 at the 3 sites sampled in both years (Seward, Homer, Kenai; Fig. 3). For the sites sampled in 2008 only, estimated prevalence was 0.98 (± 0.03) in Juneau, 0.76 (± 0.08) in Haines, and 0.03 (± 0.03) in Valdez (Fig. 3). We found no support for effects of sex, body condition, or co-infections on *Haemoproteus* prevalence (Additional file 1: Table S3), although at the 2 sites where *Haemoproteus* was common, all sampled juveniles were infected. Spatial heterogeneity in the occurrence of *Haemoproteus* infections precluded formal analysis, but visual examination of weather variables plotted against *Haemoproteus* prevalence did not reveal any apparent relationships (Fig. 3).

**Fig. 4:**
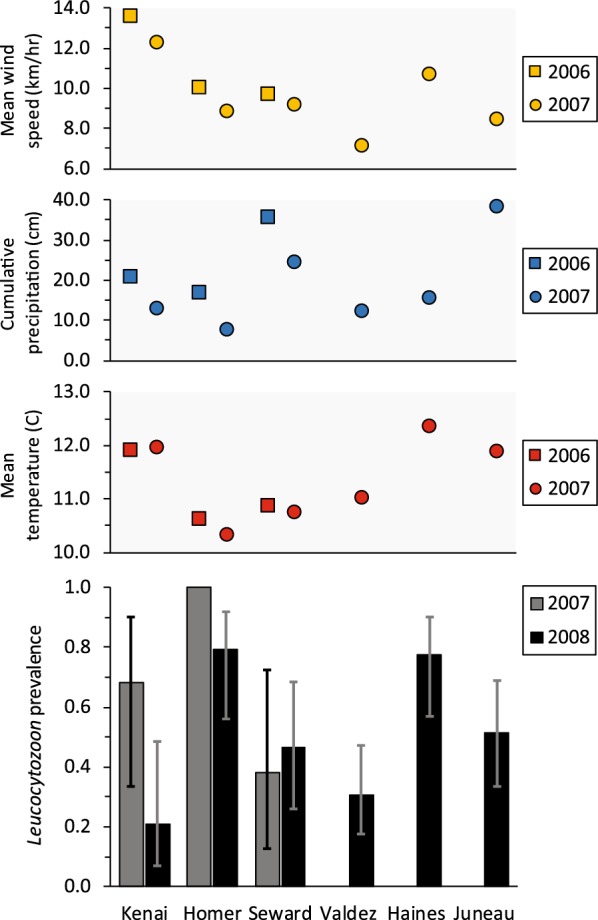
Estimated prevalence (± 95% CI) of *Leucocytozoon* parasites infecting northwestern crows (*Corvus caurinus*) sampled in late winter during 2007 (grey) and 2008 (black) at 6 sites in southcentral and southeastern Alaska. Mean hourly wind speed (yellow), cumulative precipitation (blue), and mean daily temperature (red) for summer (1 May to 31 August) during the preceding year are shown with a square (2006) or circle (2007). No relationships were apparent between parasite prevalence and any of the weather variables. Note that Valdez, Haines, and Juneau were sampled in 2008 only

*Leucocytozoon* was the most common parasite genus and infections were identified in crows at all study sites in both years (Table 1; Fig. 4). The top approximating Stage 1 model for *Leucocytozoon* allowed detection to vary by sex, and prevalence to vary by an interaction of location and year (Additional file 1: Table S4). Estimated detection probability was higher for males (0.91 ± 0.03) than females (0.76 ± 0.06). We found limited support for an age effect (ΔAIC_c_ = 1.16), with prevalence higher among adult crows, but there was no support for variation in prevalence relative to sex (Additional file 1: Table S4). Estimated *Leucocytozoon* prevalence was highest in Homer during 2007 (1.0) and lowest in Kenai during 2008 (0.21 ± 0.11; Fig. 4). For the 3 sites at which birds were sampled in both years, prevalence was relatively consistent across years at Seward (2007: 0.38 ± 0.17; 2008: 0.47 ± 0.12) and Homer (2007: 1.0; 2008: 0.79 ± 0.12) but was considerably higher at Kenai during 2007 (0.68 ± 0.16) than 2008 (0.21 ± 0.11). We found no support for relationships between *Leucocytozoon* prevalence and body condition, co-infections, or weather indices (Additional file 1: Table S4).

### Genetic diversity

We were able to assign lineages to 18/18 samples that were positive for *Plasmodium*, 46/55 samples that were positive for *Haemoproteus*, and 65/100 samples that were positive for *Leucocytozoon*. Most *Plasmodium* infections in northwestern crows were from a single lineage (NOCRP01), which was present at all three sites where *Plasmodium* was detected (Table 2). We had only a single detection of another closely related *Plasmodium* lineage (NOCRP02) at Kenai, where prevalence of *Plasmodium* was highest. NOCRH01 was the most common *Haemoproteus* lineage and occurred only at our most southerly sites (Haines and Juneau). We detected a second *Haemoproteus* lineage (NOCRH02) at low frequencies at Haines, Juneau, and Valdez. We identified six *Leucocytozoon* lineages, although only one of these (NOCRL01) occurred commonly and at all sites. Three other lineages (NOCRL04, NOCRL05, NOCRL06) were present at Haines and Juneau only; one (NOCRL02) was detected at Seward, Valdez, and Homer; and one (NOCRL03) had a single detection at Seward.

**Table 2:** Occurrence of parasite lineages detected in northwestern crows (*Corvus caurinus*) at six sites in coastal Alaska during 2007 and 2008

Parasite lineage	Kenai	Homer	Seward	Valdez	Haines	Juneau	Total
*Plasmodium*							
NOCRP01	12	4			1		17
NOCRP02	1						1
*Haemoproteus*							
NOCRH01					20	16	36
NOCRH02				1	1	8	10
*Leucocytozoon*							
NOCRL01	7	18	5	10	9	3	52
NOCRL02		2	3	1			6
NOCRL03			1				1
NOCRL04					3	1	4
NOCRL05						1	1
NOCRL06					1		1

### Phylogenetic analysis

**Fig. 5:**
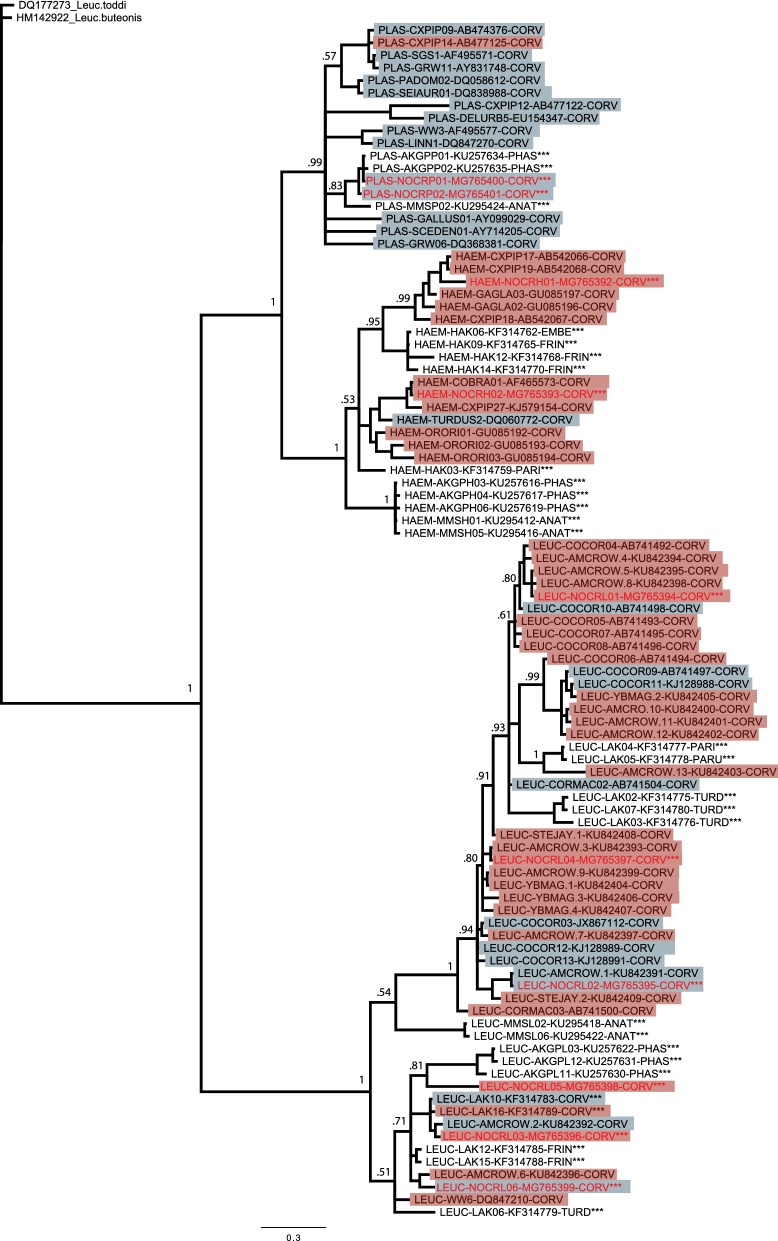
Bayesian phylogenetic tree of parasite mtDNA cytochrome *b* lineages from northwestern crows (*Corvus caurinus*) and accompanying reference lineages. Lineages isolated from samples in this study are labeled in red; lineages isolated from avian hosts in Alaska are marked with asterisks. Highlighting denotes whether a lineage found in corvid hosts is specific only to corvids (pink) or has been found in other host families (grey). Node tips are labeled with abbreviated parasite genus (PLAS, *Plasmodium*; HAEM, *Haemoproteus*; LEUC, *Leucocytozoon*) followed by the lineage name, GenBank accession number, and abbreviated family name for the avian host (CORV, Corvidae; PHAS, Phasianidae; ANAT, Anatidae; EMBE, Emberizidae; FRIN, Fringillidae; PARI, Paridae; PARU, Parulidae; TURD, Turdidae)

Parasite lineages clustered by genus as expected, with all major clades of our phylogeny receiving high posterior probability values (Fig. 5). We observed evidence for generalist behavior of *Plasmodium* parasites in northwestern crows, with both lineages (NOCRP01, NOCRP02) forming a clade with other *Plasmodium* reference sequences from Alaskan host species. The most common lineage, NOCRP01, was identical to the BT7 lineage found on the MalAvi database (identified as P43 in GenBank), which has been previously identified in over 11 host families. *Haemoproteus* lineages in northwestern crows clustered into two distinct clades, each consisting of lineages unique to corvids, excluding a single lineage found in corvids as well as nine other host families. The *Leucocytozoon* lineages we identified in our samples appeared to exhibit varying levels of specificity but lacked any distinct patterns. Two lineages (NOCRL02 and NOCRL06) clustered with those from other corvid hosts but were identical to *Leucocytozoon* lineages previously detected in other host families. The other four, while unique or identical only to other lineages detected in other corvid species, showed little evidence of any patterns of host specificity.

## Discussion

Our study examined prevalence and diversity of avian blood parasites (*Plasmodium*, *Haemoproteus*, and *Leucocytozoon*) among northwestern crows in Alaska and assessed biotic and abiotic factors that may be related to parasite infection in this northern resident host species. We found evidence for strong site-specific differences among all three genera. However, distribution of parasites did not vary along latitudinal gradients and differences between sites were not explained by the climatic factors we analyzed (wind, precipitation, or temperature). The most southerly of our sampling locations, which were also among the warmest, had low or zero prevalence of *Plasmodium*, indicating that mean summer temperature or latitude were themselves inadequate to explain variation in prevalence. Host characteristics, including age and sex, were not related to infection status in crows, which may be explained in part by the ecology of this species, as well as the timing of our sampling. Co-infections with haemosporidian parasites and AKD were common, and we detected co-infections with multiple haemosporidian genera in more than a third of infected birds. However, in contrast to other related studies [16, 19, 65], we found no evidence that infection status for a given parasite genus was correlated with AKD or the presence of other blood parasites. Occupancy modeling indicated that parasite detection probability for a single PCR run varied by genus (0.76–1.0) but was generally high, suggesting that duplicate analysis allowed us to identify almost all of the infections. Finally, we identified higher lineage diversity for *Leucocytozoon* than for *Haemoproteus* or *Plasmodium*. For select *Leucocytozoon* lineages, we also observed weaker patterns of host specificity, which is contrary to results from *Leucocytozoon* lineages identified in other avian species in Alaska, and among many corvids (see detailed discussion below).

### Spatial and temporal patterns

Northwestern crows in Alaska were commonly infected by avian blood parasites, but prevalence of each genus varied strongly by location. We detected *Plasmodium* and *Haemoproteus* sporadically across sites, whereas *Leucocytozoon* was present at all locations. We observed a year effect for *Leucocytozoon*, but not for *Plasmodium*, and *Haemoproteus* was detected exclusively at sites where we sampled in one year only. Interestingly, *Plasmodium* occurred at a higher prevalence (54%) among crows at Kenai than has been reported elsewhere for Alaskan birds but was rare or absent at our other study locations. Despite its close geographical proximity to Homer and documented movements of individual crows between the two sites (C. Van Hemert, unpublished data), prevalence of *Plasmodium* was four times higher at Kenai. *Plasmodium* is transmitted by mosquitoes (Culicidae; [1]), which are common throughout most of Alaska and occur in forested habitats adjacent to our sampling sites. However, knowledge about competent vectors in this region is extremely limited and therefore any suppositions regarding specific vector species are necessarily speculative. Characteristics of our study sites were generally similar, consisting of abundant intertidal habitat with adjacent mature spruce-hemlock forest, but microhabitat features vary across this region and could influence the abundance and diversity of vectors. For example, Kenai is near a large, tidally affected river, and coastal habitats in this area typically consist of more mud and standing fresh water than any of the other sites (C. Van Hemert, personal observations). These conditions may be conducive to mosquito production, including viable *Plasmodium* vectors. Additionally, Kenai is the most climatically similar to and geographically connected with interior boreal habitats. Other arthropod and host–parasite communities in northern regions have been shaped by historic climatological processes, including major glaciation events [66, 67]. It is likely that biogeographical factors, both contemporary and historic, have similarly contributed to the structure and connectivity of vector communities in southeastern and southcentral Alaska. Recent findings from southcentral Alaska suggest that *Culiseta* mosquitoes may be implicated in transmission of the BT7 *Plasmodium* lineage [68], which we identified in the majority of infected crows in this study. Additional research on vectors, particularly *Culiseta* mosquitoes, may help to elucidate regional and local patterns of *Plasmodium* distribution and prevalence in Alaska.

The geographical patterns of prevalence we observed for *Haemoproteus* indicate that conditions at the sites we sampled in southeastern Alaska are especially suitable for this parasite genus or its vectors. *Haemoproteus* was largely restricted to Haines and Juneau, our most southerly sites, where prevalence was 0.76 and 0.98, respectively. Interestingly, every juvenile sampled at these sites was infected with *Haemoproteus. Haemoproteus* parasites are typically transmitted by biting midges of the genus *Culicoides*, which require semi-aquatic or very moist habitats for reproduction [1, 69]. However, some of the other sites we sampled outside of southeastern Alaska also receive abundant summer rainfall, so water is unlikely to be the only limiting factor. Large geographical features such as mountain ranges and water bodies may serve as ecological barriers, potentially limiting connectivity or spread of certain vector species, although information about *Culicoides* or other potential vectors in Alaska is lacking. Additionally, historical biogeographical patterns may have contributed to the current distribution and composition of local vector communities and host–parasite systems [70]. Evidence for local transmission of *Haemoproteus* parasites in Alaska has been demonstrated in multiple species of waterfowl at 65°N [16] and infections have also been detected in resident songbirds as far north as 67° [17]; thus, the near-absence of *Haemoproteus* parasites in crows outside of southeastern Alaska was unexpected.

Finally, we detected *Leucocytozoon* in crows sampled at all six sites, but similar to that in the other parasite genera, prevalence showed considerable geographical variation, ranging from 0.21 at Kenai in 2008 to 1.0 at Homer in 2007. *Leucocytozoon* parasites are transmitted by haematophagous biting flies (Simuliidae), which are typically found at highest densities near moving water [1]. All sites we sampled had an abundance of small streams and rivers but it is unknown whether local hydrological factors influence these vector communities. We were unable to assess location-specific patterns of temporal variation because sampling occurred over relatively short time periods at each location (usually < 1 week) and locations were not sampled simultaneously. However, given that all our sampling occurred within a 2-month period during winter when vectors are inactive, it is reasonable to assume that date had a minimal effect in our study.

Contrary to our expectations, no clear explanatory patterns emerged to suggest that summer temperature, precipitation, or wind speed was responsible for the geographical or temporal variation we observed in prevalence for any of the three parasite genera. This was somewhat surprising because other studies conducted in diverse ecological settings have identified climatic variables, particularly temperature, as important determinants of parasite prevalence [13, 19, 71]. Similarly to ours, these studies sampled birds across sites with varying mean temperatures, wind speed, and annual precipitation, although in some cases the distances between sites were relatively small. Overall, our sites covered a broad geographical area, with nearly 1000 km separating our most easterly and westerly sites, and showed considerable variation in weather parameters; mean summer temperature varied by ~ 2 °C (10.3–12.4 °C) and there was a nearly two-fold difference in wind speed and a five-fold difference in rainfall across sites (Figs. 2, 3, 4). As a general rule, *Plasmodium* parasites are more sensitive to changes in temperature than *Haemoproteus* or *Leucocytozoon* species [1], and while the site with the highest prevalence (Kenai) was one of the warmest, both sites in southeastern Alaska had similar temperatures but no or very few *Plasmodium* infections. Additionally, the site with the second-highest estimated prevalence (Homer) had the lowest mean temperature in both years (Fig. 2). It is possible that a 2 °C difference in mean temperature was not of sufficient magnitude to influence parasite prevalence, although periods of active transmission may be associated with modest temperature increases in high latitude environments [1]. For example, *P. relictum* requires a minimum temperature of 13 °C for sporogonic development, which exceeds the mean temperatures recorded at all of our study sites during the preceding summer [59]. Small differences in daily temperature near this threshold may have a relatively large influence on parasite prevalence. Prevalence of *Haemoproteus*, which is transmitted by biting midges that require moist habitats, showed no clear relationship with any of the climatic variables we considered, including precipitation (Fig. 3). Wind speed has been correlated with prevalence of *Leucocytozoon* infection in other systems, with higher wind speeds typically resulting in lower infection rate [72], but we saw no evidence of such a relationship at our sites. Annual variation in site-specific estimates of *Leucocytozoon* prevalence were characterized by a slight increase from 2007 (0.38) to 2008 (0.47) at Seward, but prevalence at both Homer (2007: 1.0%, 2008: 0.79%) and Kenai (2007: 0.68%, 2008: 0.21%) were higher in 2007 than 2008 (Fig. 4). Annual weather indices at these three sites showed relatively similar patterns of less wind and lower levels of precipitation in 2008 *vs* 2007, and there were only minor site-specific changes in mean temperature between years, suggesting that variability in these parameters was not responsible for observed annual differences in parasite prevalence.

Given the inability of climatic variables to explain patterns of geographical and annual variation in this study, it appears as if other factors that we did not measure were the primary drivers of parasite prevalence. However, it is also plausible that temperature, wind speed, and precipitation may have contributed to variation in prevalence, but that the variables we constructed did not capture the relative effects of these factors. We compiled climatic data across the entire summer (mean hourly temperature, mean hourly wind speed, and cumulative rainfall) and although such predictors have proven useful at explaining variation in prevalence in other studies [18, 19], summary measures may not be the best predictors, particularly at sites where climatic factors straddle the threshold for parasite development or transmission. For instance, isolated periods of relatively warm weather, necessary to promote sporogonic development of *Plasmodium* parasites, or diurnal temperature ranges may be more relevant than overall mean temperature at a given site [73, 74].

The lack of correlation between blood parasite prevalence and standard weather variables suggests that model projections based solely on latitude or broad-scale climatic factors may not be informative at a regional scale. In northern regions, where large distances and remoteness limit field sampling, current and projected parasite distributions are sometimes assessed by ecoregion or other coarse landscape-level features [17, 21]. Such categorization, while useful as a starting point, may not be sensitive enough to accurately capture the conditions necessary for *Plasmodium, Haemoproteus*, or *Leucocytozoon* to be established and maintained in local hosts. A better understanding of vector populations and host–parasite dynamics in northern regions is needed to identify local drivers of parasite transmission and predict where haemosporidian parasites may occur in the future.

### Interspecific comparisons

Similar to our findings in northwestern crows, corvids in other parts of the world are frequently infected with blood parasites, particularly *Leucocytozoon*, which sometimes exceeds 90% prevalence [75, 76]. Recent studies of the closely related American crow in California detected *Leucocytozoon* in 57% of local birds, including adults and nestlings [77]; among nestlings only, prevalence was 44% for *Leucocytozoon*, 26% for *Haemoproteus*, and 18% for *Plasmodium* [41]. Steller’s jays (*Cyanocitta stelleri*) and black-billed magpies (*Pica nuttalli*) from California were also infected with *Leucocytozoon* at 57% and 55% prevalence, respectively [77]. Elsewhere in North America, *Leucocytozoon*, *Haemoproteus*, and *Plasmodium* have been detected in common ravens (*Corvus corax*; summarized in [78]) and *Haemoproteus* and *Plasmodium* have been reported in fish crows (*Corvus ossifragus*; [79]). Information about avian blood parasites in other corvids from Alaska is limited, but *Leucocytozoon* was detected in a small number of Canada jays (*Perisoreus canadensis*) sampled from the interior of the state [17].

Geographical differences in haemosporidian prevalence, such as what we observed in Alaskan crows, are also apparently common among other corvid species worldwide. For example, in Hokkaido, Japan, carrion (*C. corone*) and large-billed (*C. macrorhynchos*) crows had high prevalence of *Leucocytozoon* (> 95%), but no infections of *Haemoproteus* or *Plasmodium* were detected [75]. Investigations of the same species in southern Japan reported prevalence of 36–39% for *Leucocytozoon*, 10–12% for *Haemoproteus*, and 0–15% for *Plasmodium* [40]. Large-billed crows have been identified as a primary reservoir of *Plasmodium* lineages in some parts of Japan [26]. Studies of American crows in North America have reported a wide range of infection rates among the three parasite genera: *Leucocytozoon* (1–100%); *Haemoproteus* (0–100%); and *Plasmodium* (1–33%) (summarized in [78]). Site-specific differences were similarly observed among hooded crows (*C. corone cornix*) in Italy [76].

### Individual host factors

Specific host factors, including age and sex, were not important predictors of haemosporidian parasite prevalence in northwestern crows. Adult birds often have higher prevalence of blood parasite infection than juveniles, a pattern commonly attributed to longer cumulative exposure [1]. However, some studies, like ours, have found no discernible effect of age on parasite prevalence, e.g. [80]. All of the juvenile birds we sampled were at least nine months old and would have been exposed to potential vectors during most of the previous summer. Thus, it is possible that effects of age due to cumulative exposure may be negligible by late winter and spring, when our sampling occurred. We also found no evidence to support differences in parasite prevalence between male and female crows. Northwestern crows employ bi-parental care, with both parents attending the nest and provisioning nestlings, and show limited sexual segregation throughout the annual cycle [24], so it is perhaps not surprising that their exposure to vectors would be similar.

In contrast to results from a similar study of *Plasmodium* in black-capped chickadees [19], we did not detect a relationship between clinical signs of AKD and infection with avian blood parasites. Birds with AKD, which is suspected to be caused by a viral pathogen [81], exhibit gross beak deformities that compromise their ability to feed, preen, and conduct other necessary functions [28, 29, 82] and we expected that behavioral or immune-related differences might influence parasite prevalence. However, there may be other factors unrelated to AKD, such as nest-site selection, that are stronger determinants of the likelihood of parasite infection than individual health or disease state. Given the relatively low prevalence of both AKD and *Plasmodium* in our study, it is possible that we lacked the resolution to detect such synergistic effects. Alternatively, if co-infection resulted in high mortality, affected individuals would not be available to be sampled.

For northwestern crows, our results also suggest that infection with a parasite of a given genus occurs independently of infections with parasites of other genera. In contrast, several previous studies have identified relationships between *Leucocytozoon* and *Haemoproteus* in co-infection; in most cases, a positive correlation was reported [16, 83], although the converse has also been observed among passerines in Alaska [17]. The co-occurrence of multiple parasite genera within a single host has been explained by a variety of proposed mechanisms, including effects on immunocompetence [4, 35], within-host parasite competition [17], and common vectors or shared vector habitat [16]. One study suggested that infection with another parasite may be a prerequisite for infection by *Leucocytozoon* [84]. Given that we observed relatively high *Leucocytozoon* prevalence in the absence of other parasites, it seems unlikely that such a requirement exists in northwestern crows.

We did not detect a relationship between size-adjusted mass and infection status for *Plasmodium*, *Haemoproteus*, or *Leucocytozoon* parasites in northwestern crows, suggesting that probability of infection was independent of the host’s body condition. The effects of haemosporidian parasites on birds are known to vary by parasite genus, with *Plasmodium* most clearly linked to pathogenicity in other corvid species, particularly among native Hawaiian crows [6, 9, 40]. Given that northwestern crows likely have a long-standing evolutionary relationship with *Plasmodium*, it is plausible that deleterious effects of chronic infection are minimal or nonexistent. However, Townsend et al. [41] recently showed that infection of nestling American crows by *Plasmodium* parasites (but not *Leucocytozoon* or *Haemoproteus* parasites) was associated with reduced fledging success and apparent survival during the first 3 years of a bird’s life. *Plasmodium* parasites were the least commonly detected among the three genera in our study and occurred at a relatively high prevalence at only one site. Thus, we may have lacked the statistical power to detect an effect of body condition because of small sample sizes and possible confounding with site effects.

Reduced body condition may also influence an individual’s susceptibility to blood parasite infection or recrudescence of latent infections, such as has been reported for *Haemoproteus*/*Plasmodium* among lesser scaup *Aythya affinis* [35]. Yet such relationships do not appear to be uniform across species or host–parasite systems. Like ours, a number of other studies have detected no or minimal support for a correlation between body condition and blood parasite infection [83, 85, 86]. It is also important to note that we captured birds during late winter, and infections likely occurred at least six months prior when vectors in the area are typically active. Thus, whereas our results suggest that body condition during late winter is not related to probability of infection during the preceding summer, we are unable to provide inference on such relationships during the acute phase of infection.

### Parasite diversity and host conservatism

As expected, patterns of genetic diversity and host specificity among crows varied by parasite genus. We detected fewer lineages of *Plasmodium* and *Haemoproteus* parasites in our samples, with only two lineages each. *Leucocytozoon* in crows was much more diverse, although, as was the case for the other two genera, a single lineage comprised most of the infections. Our findings concur with previous studies of corvids and other avian hosts, which generally report higher lineage diversity for *Leucocytozoon* than other parasite genera [77, 87].

*Plasmodium* lineages showed typical generalist behavior, as none of our sequences was unique to corvids and they were most closely related to lineages identified in taxonomically diverse species, including tetraonids (grouse and ptarmigan), passerines, and waterfowl sampled in Alaska (Fig. 5). In fact, the BT7 lineage of *Plasmodium* that we identified most commonly in crows has also been documented in a variety of avian taxa across Alaska [15, 17, 19, 87], further supporting the apparent generalist behavior of *Plasmodium* in this system. In contrast, *Haemoproteus* lineages exhibited higher levels of host conservatism than *Plasmodium*, a trend that has been suggested in previous studies [88, 89] and observed in multiple populations of Alaskan bird hosts [15, 87]. *Leucocytozoon* lineages, which often exhibit the strongest levels of host specificity across haemosporidian genera, showed less specificity to corvids than has been reported elsewhere in Alaska [15, 87]. A number of previous studies also reported high host conservatism for *Leucocytozoon* parasites within corvid species, although a more recent genetic analysis suggested that previous taxonomic categorizations of parasites based on morphological data alone may be inaccurate and that some *Leucocytozoon* parasites that infect corvids may in fact be generalist species [77]. The distribution of northwestern crows overlaps those of some terrestrial tetraonid, passerine, and waterfowl species during summer when vectors are likely to be actively transmitting parasites. As such, the apparent lack of host specificity of *Leucocytozoon* lineages identified in this study may be explained by vectors shared among crows and other Alaskan bird species.

## Conclusions

Our results suggest that adult and juvenile northwestern crows in Alaska are commonly infected with avian blood parasites, although genus-specific prevalence varied strongly among sites, including those with similar habitat and environmental conditions. Whereas our indices of summer temperature, wind speed, and cumulative precipitation also varied considerably among sites, we found no relationship between parasite prevalence and any of these climatic variables. As such, we advise that caution is warranted when making large-scale projections about current or future parasite distributions based on a limited number of sampling locations. Future research is needed to better understand specific drivers of infection by blood parasites in northern hosts, particularly relative to vector populations, which have not been well described in this region. We observed no relationship between host body condition and parasite infection, nor patterns of co-infection relevant to individual health. Additional research examining the fitness consequences of infection among haemosporidian-naïve birds that occur in the Arctic and sub-Arctic would help to determine whether potential changes in vector communities or parasite distribution may pose a threat to resident avian populations.

## Additional file

**Additional file 1: Table S1.** A summary of the occupancy modeling approach for estimating probability of haemosporidian parasite infection and probability of detecting haemosporidian mtDNA in northwestern crows (*Corvus caurinus*) relative to temporal, spatial, and host characteristics. Models are ranked by ΔAIC_c_ values. **Table S2.** AIC_c_ model-selection results for *Plasmodium* infection in northwestern crows (*Corvus caurinus*) relative to temporal, spatial, and host characteristics. **Table S3.** AIC_c_ model-selection results for *Haemoproteus* infection in northwestern crows (*Corvus caurinus*) relative to temporal, spatial, and host characteristics. **Table S4.** AIC_c_ model-selection results for *Leucocytozoon* infection in northwestern crows (*Corvus caurinus*) relative to temporal, spatial, and host characteristics.

## Data Availability

The dataset supporting the conclusions of this article is available in the USGS Alaska Science Center repository (10.5066/P9EBB1LG [90]).

## Abbreviations

AIC: Akaike’s information criterion
AKD: avian keratin disorder
BCI: body condition index
